# Alginate/PVA Hydrogel Incorporating HA-Liposomes and Aronia-Derived Silver Nanoparticles for Advanced Wound Management

**DOI:** 10.3390/ijms26189203

**Published:** 2025-09-20

**Authors:** Anca-Elena Țăin (Anastasiu), Alexandra Cătălina Bîrcă, Ana Maria Isabela Naulea, Adelina-Gabriela Niculescu, Alexandru Mihai Grumezescu, George-Alexandru Croitoru

**Affiliations:** 1 Department of Science and Engineering of Oxide Materials and Nanomaterials, National University of Science and Technology POLITEHNICA Bucharest, 011061 Bucharest, Romania; anca_tain@yahoo.com (A.-E.Ț.); ada_birca@yahoo.com (A.C.B.); aminaulea@gmail.com (A.M.I.N.); adelina.niculescu@upb.ro (A.-G.N.); 2 Research Institute of the University of Bucharest—ICUB, University of Bucharest, 050657 Bucharest, Romania; 3 Faculty of Dental Medicine, Carol Davila University of Medicine and Pharmacy, 8 Eroii Sanitari Street, 050474 Bucharest, Romania; alex.croitoru@umfcd.ro

**Keywords:** hydrogels, bilayer wound dressings, wound healing, tissue regeneration, poly(vinyl alcohol), alginate, liposomes, hyaluronic acid, silver nanoparticles, aronia reducing agent

## Abstract

Chronic wounds remain a persistent clinical challenge due to delayed healing, recurrent infections, and limited effectiveness of conventional dressings. To address these unmet needs, we designed a multifunctional hydrogel system based on poly(vinyl alcohol) (PVA) and alginate (ALG), incorporating hyaluronic acid (HA)-loaded dipalmitoylphosphatidylcholine (DPPC) liposomes for regenerative stimulation and Aronia-mediated silver nanoparticles (Ag_Aro) for antimicrobial protection. Physicochemical analyses (DLS, SEM, FTIR) confirmed the successful assembly of the system and demonstrated distinct particle sizes, pore morphologies, and structural interactions. Swelling and degradation studies revealed favorable hydration capacity and stability under physiologically relevant conditions. In vitro assays with HaCaT keratinocytes indicated excellent biocompatibility, with HA-liposomes enhancing cell viability to ~190% and Ag_Aro showing minimal cytotoxicity, likely due to polyphenolic surface capping. The combined formulation achieved a balanced swelling profile, controlled degradation, and the highest biocompatibility (~195% viability), underscoring the synergistic benefits of the dual-agent design. This study introduces, to our knowledge, the first PVA–ALG bilayer hydrogel integrating HA-liposomes and phytosynthesized AgNPs, offering a promising platform for advanced wound management. Further in vivo studies are warranted to validate its therapeutic performance.

## 1. Introduction

Wound healing supposes a multifaceted and tightly controlled physiological process for restoring skin integrity following injury [1]. Acute wounds follow an organized healing trajectory culminating in complete tissue regeneration. However, improper management or underlying comorbidities can disrupt the healing process, leading to the formation of chronic wounds [2,3,4]. More specifically, chronic wounds are defined as skin injuries that fail to progress through the normal healing stages within 4–6 weeks and often require over 1–3 months for functional and anatomical recovery [5,6,7,8]. These wounds are frequently associated with systemic pathologies such as diabetes mellitus, vascular insufficiency, or prolonged pressure, being clinically represented by diabetic foot ulcers, pressure sores, and deep burns [6,9,10]. The chronic wound microenvironment is characterized by persistent inflammation, impaired vascularization, and dysregulated immune responses, which collectively delay re-epithelialization and increase susceptibility to infection [8,11,12]. Beyond the physiological burden, chronic wounds significantly contribute to healthcare costs and profoundly impact patients’ quality of life, necessitating multidisciplinary approaches for successful long-term management [3,7,13,14].

Despite their widespread clinical use, conventional wound dressings, such as gauze, cotton wool, and bandages, offer limited therapeutic benefits beyond basic protection. These passive materials primarily serve as physical barriers to environmental contaminants and allow partial evaporation of wound exudates [3,15,16]. However, they lack bioactivity, have poor moisture retention or absorptive capacity, and require frequent changes, which can disrupt the healing process and exacerbate patient discomfort. Moreover, traditional dressings do not adequately address the complex biological needs of chronic wounds, such as infection control, immune modulation, and tissue regeneration [9,15]. These limitations have prompted the development of advanced wound care strategies that incorporate bioactive compounds, antimicrobial agents, and stimuli-responsive materials to promote tissue repair and prevent complications actively [16,17].

Hydrogels have emerged as one of the most promising classes of wound dressings. They are recognized especially for their outstanding moisture-retention capacity, soothing properties, and ability to mimic the extracellular matrix (ECM). These three-dimensional, hydrophilic polymer networks can absorb high amounts of wound exudate while maintaining a moist microenvironment, which is essential for accelerating re-epithelialization and tissue regeneration [12,18,19]. Both natural and synthetic hydrogels present distinct advantages. Natural polymers (e.g., alginate, chitosan, and hyaluronic acid) are favored for their biocompatibility, hemostatic properties, and biodegradability. On the other hand, synthetic polymers like polyvinyl alcohol (PVA) offer superior mechanical robustness and physicochemical stability [9,20,21].

Alginate-based matrices effectively promote tissue repair while enabling the delivery of bioactive compounds, and PVA hydrogels provide enhanced structural support and oxygen permeability, although they often require antimicrobial augmentation [11,21]. Thus, to reconcile these features, bilayer hydrogel architectures have been increasingly explored, offering spatial separation of functionalities and the ability to incorporate different therapeutic agents in a modular manner. This dual-layer approach allows for the simultaneous promotion of regeneration and protection against microbial invasion, making it an ideal platform for advanced wound care [22]. Moreover, layered hydrogel-based systems offer enhanced versatility for further functionalization toward improving their healing performance.

Hyaluronic acid (HA) is a naturally occurring, non-sulfated glycosaminoglycan (GAG) widely distributed in connective, epithelial, and neural tissues of both vertebrate organisms and microbial sources [23]. Unlike other GAGs, HA does not form covalent bonds with core proteins, but rather exerts its biological effects through interactions with multiple cell surface receptors, including CD44 and Toll-like receptors (TLRs). This further influences a broad spectrum of cellular processes, including proliferation, migration, and immune modulation [24]. Its highly hydrophilic nature, attributed to numerous hydroxyl groups capable of forming hydrogen bonds with water molecules, makes HA an excellent candidate for maintaining tissue hydration and osmotic balance, particularly during wound healing and inflammatory responses [18]. Moreover, HA accumulates in early wound stages to support cell proliferation and migration, then undergoes enzymatic degradation as the granulation tissue matures, facilitating proteoglycan synthesis and enhancing structural resilience [23]. Additionally, the polymer’s biocompatibility, biodegradability, and structural versatility, along with its chemical modifiability, render it a highly adaptable platform for developing targeted and biomimetic biomedical materials, particularly in regenerative medicine and advanced wound care formulations [24,25].

Liposomal encapsulation has emerged as a promising strategy for enhancing the therapeutic potential of bioactive compounds, including hyaluronic acid, in wound healing applications [26]. Their structural versatility, excellent biocompatibility, and capacity for controlled release make liposomes particularly attractive for the localized and sustained delivery of both hydrophobic and hydrophilic therapeutic agents [27,28]. In the context of wound care, liposomal systems improve the pharmacokinetic profile of encapsulated substances while also reducing systemic toxicity by allowing lower effective doses [29]. Moreover, the incorporation of hyaluronic acid into liposomal carriers can synergistically enhance tissue hydration, modulate inflammation, and support cellular regeneration at the wound site. In addition, liposomes have shown significant antibacterial potential, making them suitable platforms for managing infected wounds and promoting healing in challenging clinical settings [30]. The physicochemical properties of liposomes, including size, surface charge, and colloidal stability, can be finely tuned by manipulating the lipid composition. For example, the use of cationic lipids or surfactants can alter bilayer organization and enhance interaction with negatively charged microbial membranes or cellular targets [31]. The use of dipalmitoylphosphatidylcholine (DPPC) can be a valuable option, as this amphiphilic phospholipid can spontaneously assemble into vesicular structures under ultrasound stimulation. Thus, its favorable self-assembling behavior and biocompatibility render DPPC particularly suitable for developing liposomal formulations aimed at delivering hyaluronic acid in a controlled and bioactive manner [27].

Nanotechnology has also gained traction for use in various biomedical applications. A plethora of nanomaterials have been investigated for their potential in delivering therapeutics, enhancing wound healing outcomes, and preventing/treating infections [32,33]. Particularly, silver nanoparticles (AgNPs) are widely investigated due to their broad-spectrum antimicrobial, antiviral, antifungal, and anti-inflammatory properties, which make them highly attractive for advanced therapeutic strategies, particularly in the treatment of chronic and infected wounds [34,35]. The antibacterial action of AgNPs is multifaceted, involving disruption of the bacterial membrane integrity, generation of reactive oxygen species (ROS), interaction with thiol-containing proteins, and interference with DNA replication processes [36]. AgNPs exert their antimicrobial effects through mechanisms distinct from those of ionic silver, often causing morphological and functional alterations in microbial cells, such as potassium ion leakage and cytoplasmic membrane detachment, which culminate in microbial death. However, concerns regarding their potential cytotoxicity and accumulation in vital organs have prompted an increasing interest in the development of safer, more biocompatible synthesis approaches [34,35].

Green synthesis of AgNPs using plant-derived extracts has emerged as an environmentally friendly and biologically safe alternative to conventional chemical and physical methods. This approach involves the use of phytochemicals, like flavonoids, phenols, and terpenoids, as natural reducing and stabilizing agents, enabling the conversion of silver ions into stable nanoparticles within a biologically active colloidal matrix [37,38]. The simplicity, cost-effectiveness, and eco-compatibility of plant-mediated synthesis have positioned it as a preferred strategy, with increasing evidence supporting the efficacy of green-synthesized AgNPs in preventing biofilm formation and combating drug-resistant microbial strains [39,40]. Moreover, the biomolecular corona created by plant metabolites may enhance the biocompatibility and therapeutic profile of AgNPs, while mitigating the risk of adverse cytotoxic effects [41,42,43]. As such, phytosynthesized AgNPs offer a promising platform for the development of next-generation antimicrobial wound dressings that align with both therapeutic and sustainability criteria.

Building upon these considerations, the present study proposes a multifunctional bilayer hydrogel system that integrates both regenerative and antimicrobial functionalities in a spatially organized manner. The synthetic PVA layer was selected for its structural robustness and oxygen permeability, while also serving as a carrier for HA-loaded liposomes to stimulate keratinocyte proliferation and tissue regeneration. Complementarily, the natural alginate layer provides intrinsic biocompatibility and acts as a reservoir for phytosynthesized silver nanoparticles, which ensure antimicrobial protection through broad-spectrum bactericidal activity. The bilayer configuration enables the spatial separation of these functionalities, thereby reducing potential interference between bioactive agents while maintaining their individual therapeutic benefits. To our knowledge, the combination of these components within a spatially organized bilayer PVA–alginate hydrogel matrix has not been previously reported. This study also comprehensively characterizes the physicochemical properties, swelling behavior, and in vitro biocompatibility of the developed formulations, highlighting their promise as multifunctional dressings for advanced wound therapy.

## 2. Results

### 2.1. Characterization of DPPC-Based Liposomes

**Figure 1:**
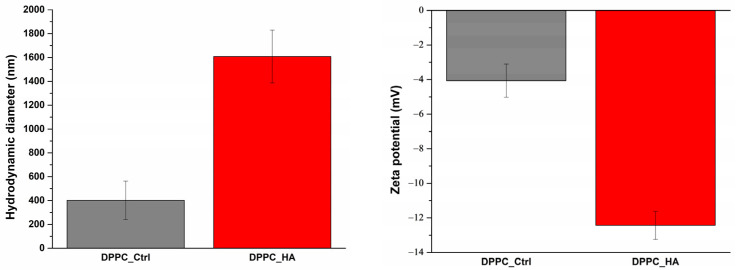
DLS analysis results for DPPC_Ctrl and DPPC_HA samples: (**left**) hydrodynamic diameter, and (**right**) zeta potential.

The physicochemical characterization of the control (DPPC_Ctrl) and hyaluronic acid-loaded (DPPC_HA) liposomes began with DLS investigations, as presented in Figure 1. DLS results revealed a mean hydrodynamic diameter of 401.93 nm for the control liposome sample, DPPC_Ctrl. Upon incorporation of hyaluronic acid, the hydrodynamic diameter increased markedly, reaching a mean value of 1608.63 nm in the DPPC_HA sample. The reported values were derived from intensity-weighted distributions, which are more sensitive to larger particles and aggregates. Polydispersity index (PDI) values were not systematically analyzed in this work, which represents a limitation for fully characterizing size heterogeneity. The pronounced increase in hydrodynamic diameter observed for HA-containing vesicles indicates that hyaluronic acid influences vesicle organization in aqueous suspension. Previous studies have shown that the presence of HA on the liposome surface increases particle size, primarily due to electrostatic interactions between HA and lipid headgroups, hydration of the polysaccharide chains, and alterations of colloidal organization [44,45,46]. Such changes contribute to the larger apparent size measured by DLS in our formulations.

Another essential parameter evaluated by the DLS technique is the zeta potential. For the DPPC_Ctrl sample, a mean zeta potential of −4.06 mV was recorded, suggesting a low colloidal stability. In contrast, the DPPC_HA sample exhibited a more negative zeta potential of −12.43 mV. This increase in negative surface charge can be attributed to the anionic character of hyaluronic acid, which contains carboxyl and hydroxyl groups capable of interacting with the lipid bilayer. Such interactions may occur either through partial encapsulation inside the vesicles or through adsorption at their surface. In both cases, the additional surface charge contributes to enhanced colloidal stability, while the presence of HA also supports specific binding to CD44 receptors on keratinocytes, promoting targeted uptake via receptor-mediated endocytosis [47,48,49,50].

**Figure 2:**
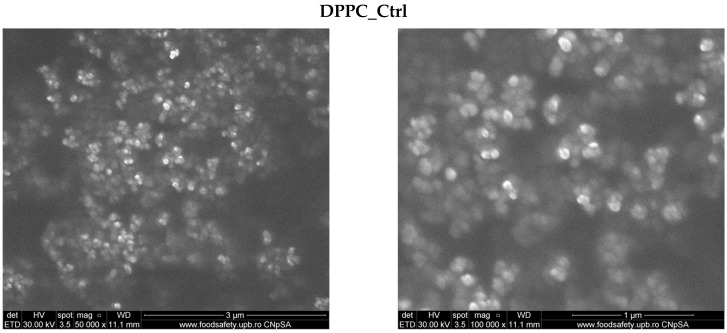

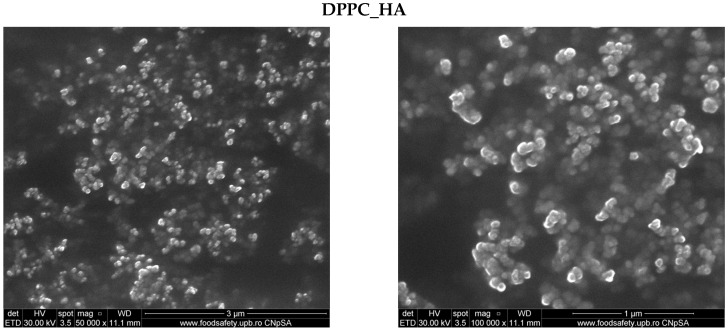
SEM micrographs of DPPC_Ctrl and DPPC_HA samples at 50,000× and 100,000× magnifications.

SEM images (Figure 2) illustrate vesicle-like domains for both the DPPC_Ctrl and DPPC_HA samples. The micrographs suggest the presence of nanoscale lipid structures, although aggregation effects are evident, and resolution limitations prevent detailed morphological interpretation. While spherical features can be discerned, SEM is not ideally suited for hydrated lipid systems, and the images should be regarded as qualitative support for vesicle formation rather than definitive morphological proof.

**Figure 3:**
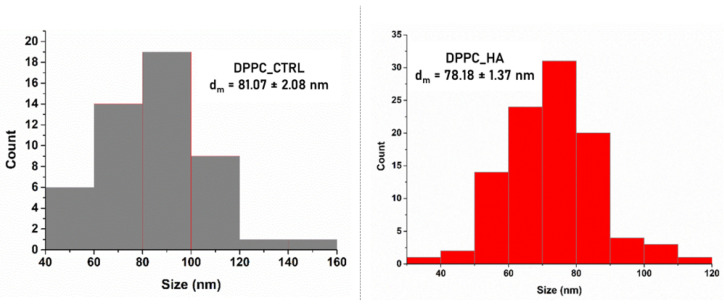
Size distribution of DPPC_Ctrl and DPPC_HA liposomes.

As expected, the apparent diameters observed by SEM (Figure 3) were noticeably smaller than the hydrodynamic sizes measured by DLS, since DLS records hydrated vesicles in suspension, which may include swelling or transient aggregation. At the same time, SEM captures the morphology of dehydrated and collapsed structures after sample preparation [51,52]. The hygroscopic character of HA likely accentuated solvent loss during drying, contributing to more pronounced shrinkage and aggregation in DPPC_HA [53,54,55]. Overall, these results confirm that HA incorporation modifies vesicle organization, producing larger hydrodynamic sizes in solution and enhanced aggregation effects upon dehydration.

### 2.2. Characterization of Ag_Aro Silver Nanoparticles

**Figure 4:**
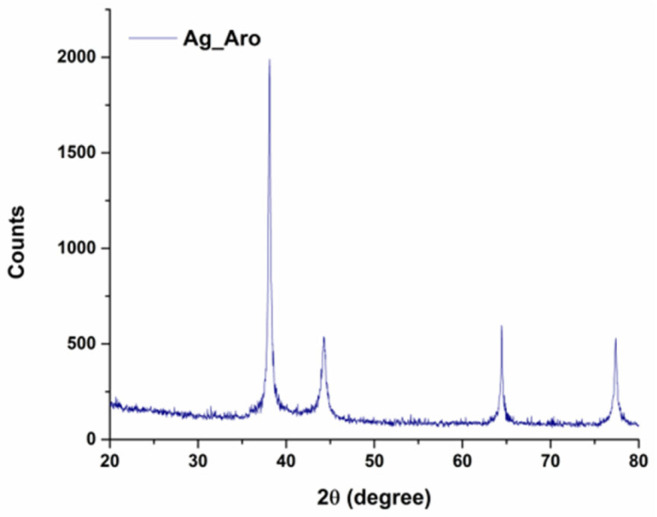
XRD diffractogram of Ag_Aro.

The green-synthesized silver nanoparticles were first characterized via XRD, as depicted in Figure 4. The obtained diffractogram confirmed the formation of pure metallic silver with a face-centered cubic (FCC) crystalline structure [56,57,58]. The diffraction peaks observed at 2θ angles of 38.09°, 44.23°, 64.45°, and 77.37° correspond to the crystallographic planes (111), (200), (220), and (311), respectively. These results align with the reference pattern in the JCPDS database (card no. 01-071-6549), affirming the structural purity of the sample. No additional peaks were detected, suggesting the absence of secondary phases or crystalline impurities. The average crystallite size was estimated to be 30.59 nm using the Debye–Scherrer equation:

$$D=\frac{K\times \lambda }{\beta \times cos\theta }$$

where D denotes the mean crystallite size, λ is the X-ray wavelength (0.1540 nm), β refers to the full width at half maximum (FWHM) of the diffraction peak, and θ is the corresponding Bragg angle.

Further, DLS analysis was performed on the silver nanoparticle sample. The investigation showed a mean hydrodynamic diameter of 212.76 nm, indicative of nanoscale particle formation. The relatively large size may be related to partial nanoparticle aggregation and/or the adsorption of phytochemicals from Aronia, which can act as reducing and stabilizing agents during synthesis. The measured zeta potential of −20.96 mV suggests favorable colloidal stability. Typically, absolute zeta potential values beyond ±30 mV indicate high electrostatic repulsion and enhanced stability [59]. Hence, while short-term stability is acceptable, long-term aggregation may still occur depending on environmental conditions such as pH and ionic strength. The negative surface charge is likely due to acidic functional groups (e.g., carboxyl and phenolic moieties) from the Aronia extract [60], which act both as reducing and stabilizing agents during nanoparticle synthesis.

**Figure 5:**
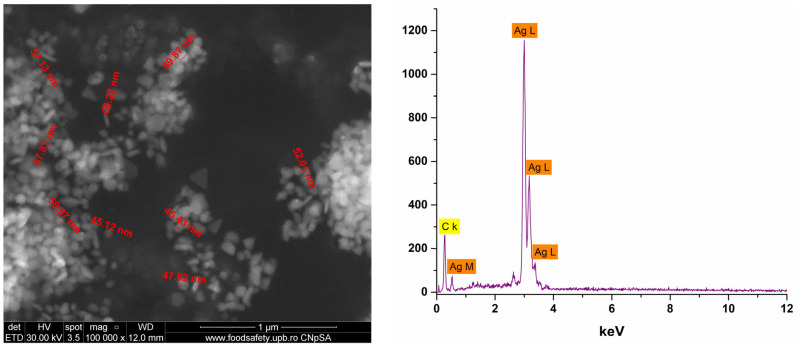
SEM micrograph and EDS results for Ag_Aro sample.

Additional SEM micrographs confirmed the nanometric scale of the Ag_Aro particles, with average dimensions ranging from 30 to 60 nm (Figure 5). The particles displayed polydisperse morphology, including spherical, quasi-spherical, triangular, truncated triangular, and elongated shapes. Such morphological diversity can impact biological activity, with truncated triangular particles offering increased surface area and potentially enhanced antimicrobial effects compared to spherical counterparts [36]. Furthermore, EDS analysis confirmed the elemental composition, identifying silver (Ag) and carbon (C). The carbon signal originates from the carbon tape used during SEM sample preparation. EDS analysis confirmed the presence of silver and a carbon background from the support tape. No clear signals from other elements were detected; however, due to its limited sensitivity for light elements (C, O, N), EDS cannot exclude the presence of organic stabilizing compounds derived from Aronia at the nanoparticle surface.

Following the successful physicochemical characterization of both DPPC-based liposomes and green-synthesized silver nanoparticles (Ag_Aro), these nanostructures were further investigated for their incorporation into hydrogel-type wound dressings.

### 2.3. Characterization of Hydrogels

**Figure 6:**
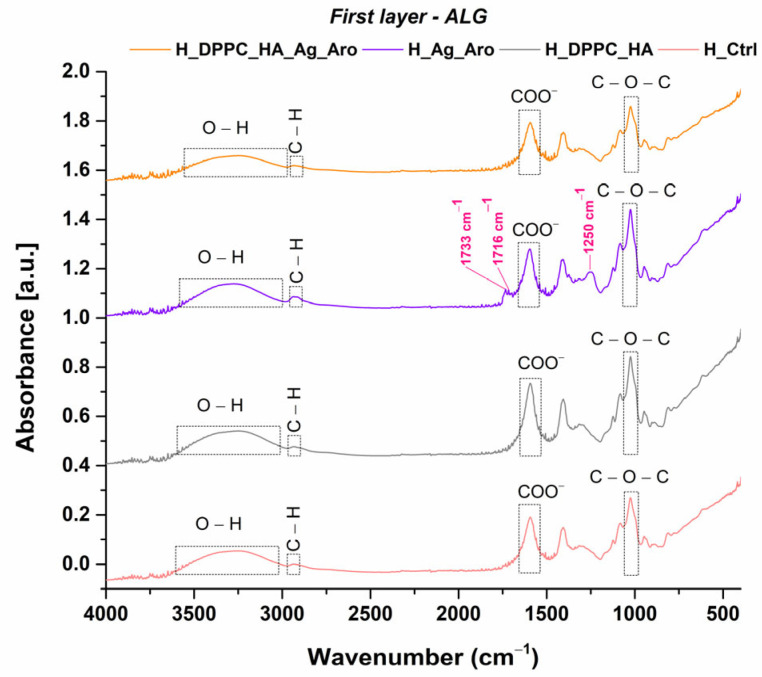
FTIR spectra of the ALG layer for each hydrogel formulation.

**Figure 7:**
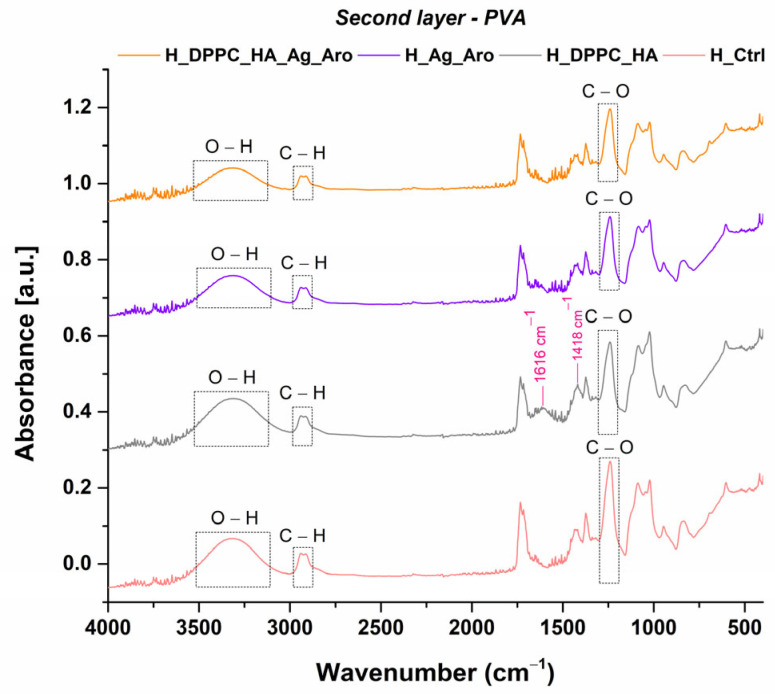
FTIR spectra of the PVA layer for each hydrogel formulation.

FTIR spectra were recorded for each layer of every sample. Figure 6 and Figure 7 compare the results corresponding to the first ALG layer, both unmodified and modified with Ag_Aro, and the second PVA layer, both unmodified and incorporating DPPC_HA liposomes.

The FTIR spectra of the ALG layer confirm the presence of the main functional groups characteristic of this biopolymer, thereby validating the chemical integrity of the matrix. The broad band observed in the 3000–3500 cm^−1^ region corresponds to O–H stretching vibrations, indicating the presence of hydroxyl groups and potential hydrogen bonding interactions within the alginate structure [61]. The peaks in the 2918–2938 cm^−1^ range are attributed to C–H symmetric stretching vibrations from the hydrocarbon chains of alginate [61]. A strong absorption band at 1405 cm^−1^ is associated with carboxylate (COO^−^) groups, and the C–O–C asymmetric stretching is observed at 1024 cm^−1^, typical of the alginic acid polymer structure [62]. In the FTIR spectrum of the Ag_Aro-containing alginate layer (H_Ag_Aro), absorption bands at 1733 cm^−1^, 1716 cm^−1^, and 1250 cm^−1^ may be associated with polyphenolic functional groups originating from Aronia. These signals support the presence of organic moieties at the nanoparticle surface, consistent with the proposed role of phytochemicals as capping agents. However, direct quantification (e.g., by TGA) was not performed in this study. The absorption bands at 1733 cm^−1^ and 1716 cm^−1^ may also reflect non-covalent interactions between anthocyanins and the alginate matrix. The band at 1250 cm^−1^ is attributed to Amide III vibrations, typically associated with N–H deformation and C–N stretching, and may originate from protein-like compounds or phenolic-polysaccharide interactions present in the aronia-mediated silver nanoparticles alginate layer [63,64,65].

The FTIR spectra of the PVA layer exhibit characteristic bands of this polymer. The band between 3000–3500 cm^−1^ corresponds to O–H stretching vibrations, and displays intensity changes according to the characteristics of each sample. The bands in the 2918–2938 cm^−1^ region are attributed to C–H stretching vibrations [62,66]. In the H_DPPC_HA spectrum, two additional absorption bands at 1616 cm^−1^ and 1418 cm^−1^ were observed, which are absent in the control and Ag_Aro containing samples. These bands are primarily attributed to the asymmetric and symmetric stretching vibrations of carboxylate groups (COO^−^) from HA. The presence of DPPC_HA vesicles may influence the position and intensity of vibrational bands through non-covalent interactions with HA [66,67]. The band observed near 1239–1240 cm^−1^, corresponding to C–O stretching, further confirms the polymeric structure of PVA [68,69]. Compared to the control sample (H_Ctrl), the formulations containing DPPC_HA exhibit intensified carbonyl and hydroxyl bands, indicating successful incorporation of these bioactive components.

**Figure 8:**
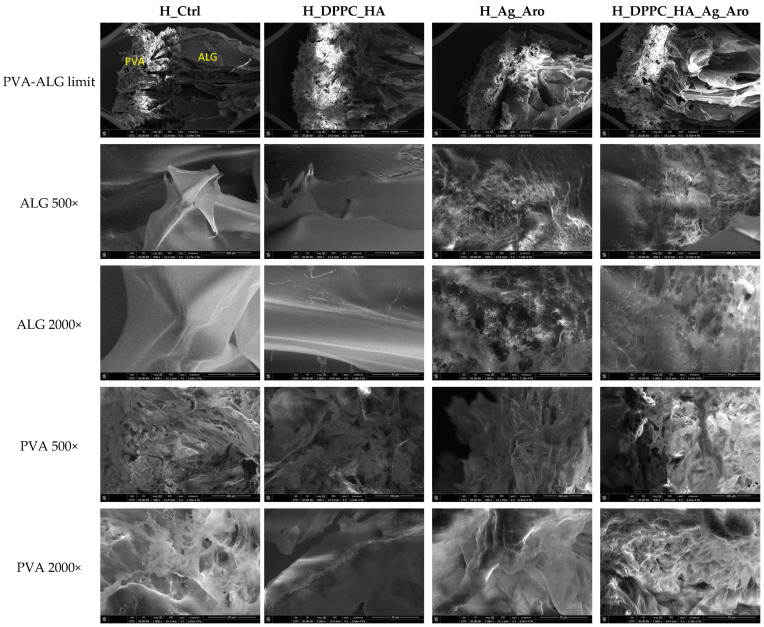
SEM micrographs obtained for H_Ctrl, H_DPPC_HA, H_Ag_Aro, and H_DPPC_HA_Ag_Aro performed at the delimitation of polymer layers and on each layer at two magnifications (500× and 2000×).

**Figure 9:**
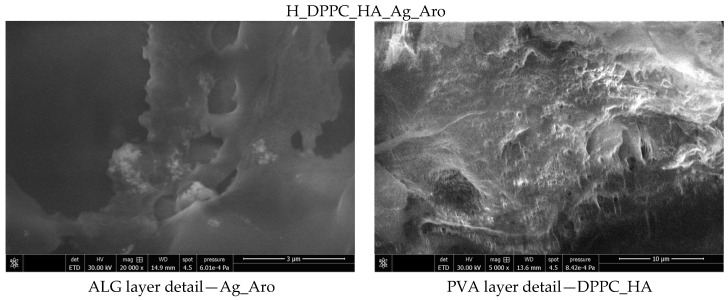
SEM micrographs obtained for H_DPPC_HA_Ag_Aro dressing at higher magnifications clearly show the presence of dual active agents.

To evaluate surface morphology and to identify the distribution of nanoparticles within the polymer matrix, the hydrogel dressings were further subjected to SEM investigations (Figure 8 and Figure 9). The obtained micrographs revealed that all four hydrogels share a porous structure, which is critical for effective wound healing. Clear delineation between the ALG and PVA layers is evident, each displaying distinct polymer arrangements and resulting in different types of porous architectures. Specifically, the ALG-based layers exhibit a longitudinal alignment of the polymer sheets, which leads to elongated pore structures. In contrast, the PVA layers appear more compact, showing a denser distribution of smaller pores.

In the case of the H_DPPC_HA formulation, the ALG layer shows similarities to that of H_Ctrl, considering that both layers are free of any nanoparticles. On the other hand, in the PVA layer containing the DPPC_HA lipid vesicles, at 2000× magnification, certain voids can be observed, which were most likely formed due to the presence of these vesicles. The H_Ag_Aro dressing reveals the presence of silver nanoparticles through the markedly different morphology of the ALG layer compared to H_Ctrl and H_DPPC_HA. These nanoparticles are distributed within the dressing layers and contribute to the formation of additional pores compared to the arrangement observed in ALG layers without Ag_Aro. Regarding the dual active-agent formulation, H_DPPC_HA_Ag_Aro, structural differences induced by the addition of active agents in both polymeric layers of the dressing are evident. Figure 9 presents higher-magnification micrographs, which allow for easier visualization of the Ag_Aro nanoparticles, homogeneously dispersed but also forming agglomerates within the dressing layers. Additionally, predominantly spherical voids can be observed in the PVA layer, which can be associated with the presence of DPPC_HA vesicles embedded in the structure of the dressing.

**Figure 10:**
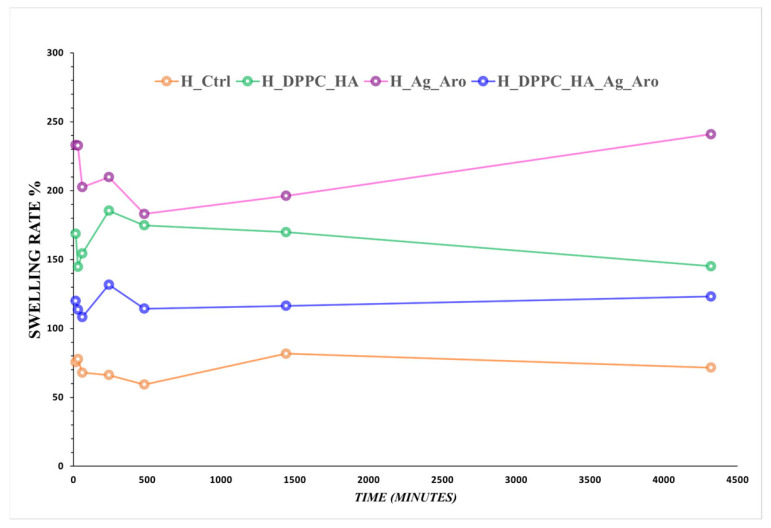
Time-dependent swelling rate of the obtained hydrogels.

The ability of the hydrogel dressings to swell in aqueous environments is of paramount importance in the development of ideal wound care materials, as this property enables the absorption of wound exudates [70]. The results obtained from this swelling study are comparatively displayed in Figure 10. Formulation H_Ag_Aro, which contains silver nanoparticles embedded in the alginate layer, exhibits the highest swelling rate across all measured time intervals. This enhanced swelling may result from the nanoparticles’ ability to reorganize the polymeric network, increasing porosity and facilitating greater water diffusion throughout the matrix [71,72]. The H_DPPC_HA formulation exhibited a moderate but gradually decreasing swelling trend, indicating a more compact polymer network that may be attributed to interactions between DPPC_HA and the polymeric matrix. The H_DPPC_HA_Ag_Aro formulation, containing both Ag_Aro nanoparticles and DPPC_HA liposomes, demonstrates a lower but consistently stable swelling profile. This behavior may reflect a more structured hydrogel architecture, which does not show significant variations in swelling behavior over an extended period of time. The reduced swelling observed in H_DPPC_HA and H_DPPC_HA_Ag_Aro may be explained by the compressive effect of the liposomes within the polymer matrix, potentially decreasing the initial porosity [73]. Conversely, H_Ctrl formulation showed the lowest swelling rates across all time points, as expected for a formulation lacking active agents that could modify network structure.

**Figure 11:**
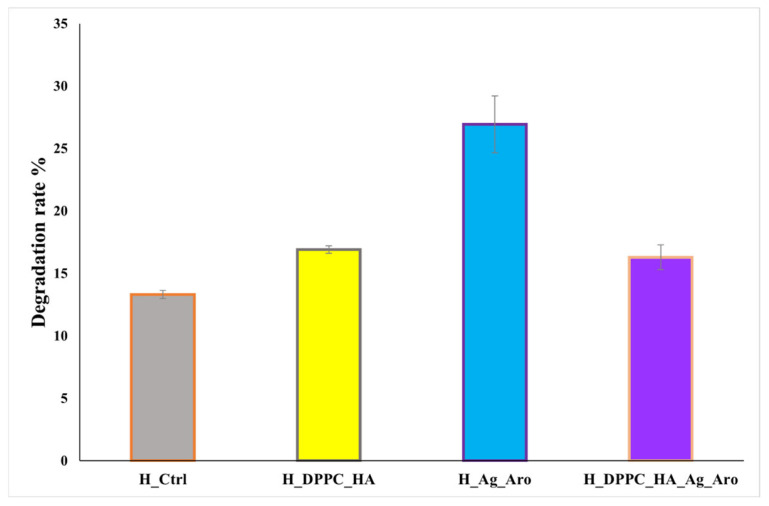
Degradation rate of the hydrogels after 72 h of PBS immersion.

Another crucial feature for hydrogels is their degradation rate. In this respect, the degradation behavior of all developed hydrogels was evaluated after 72 h of immersion in PBS, leading to the results presented in Figure 11. As can be noted, the H_Ag_Aro formulation exhibited the highest degradation rate, which is consistent with the swelling rate evaluation, where this dressing recorded the highest values at all analyzed time points. The higher degradation rate observed for the H_Ag_Aro formulation is consistent with previous reports indicating that silver nanoparticles incorporated in hydrogel matrices are gradually released into the aqueous medium, leaving voids in the polymer network and accelerating mass loss. Similar findings have been reported for alginate–AgNP systems and other polysaccharide-based nanocomposites, where nanoparticle leaching disrupted matrix integrity and enhanced degradation kinetics [35,39,62]. The H_DPPC_HA formulation showed the next highest degradation rate. Similar to H_Ag_Aro, this aligns with its high swelling capacity, suggesting that, upon contact with the aqueous environment, the DPPC_HA lipid vesicles behave in a manner comparable to Ag_Aro nanoparticles, facilitating structural disintegration. In descending order of degradation rate, the following sample is H_DPPC_HA_Ag_Aro. Based on the results obtained for the individual active agent-containing dressings, this formulation would have been expected to exhibit the highest degradation rate. However, the data suggest a stabilizing effect when both active agents are present simultaneously, potentially resulting from interactions between the DPPC_HA lipid vesicles and Ag_Aro nanoparticles that enhance the structural integrity of the hydrogel matrix.

Overall, the results obtained from physicochemical characterizations suggest that the H_DPPC_HA_Ag_Aro hydrogel successfully combines the advantages of both bioactive components, namely Ag_Aro nanoparticles and DPPC_HA lipid vesicles, achieving a favorable balance of sustained swelling and controlled degradation. These characteristics are essential for the development of wound dressings with prolonged-release functionality.

### 2.4. Biological Assay

To evaluate the biocompatibility and potential cytotoxicity of the developed bilayer wound dressings, biological assays were conducted using HaCaT keratinocyte cells to assess the safety and cellular response to materials intended for topical applications.

**Figure 12:**
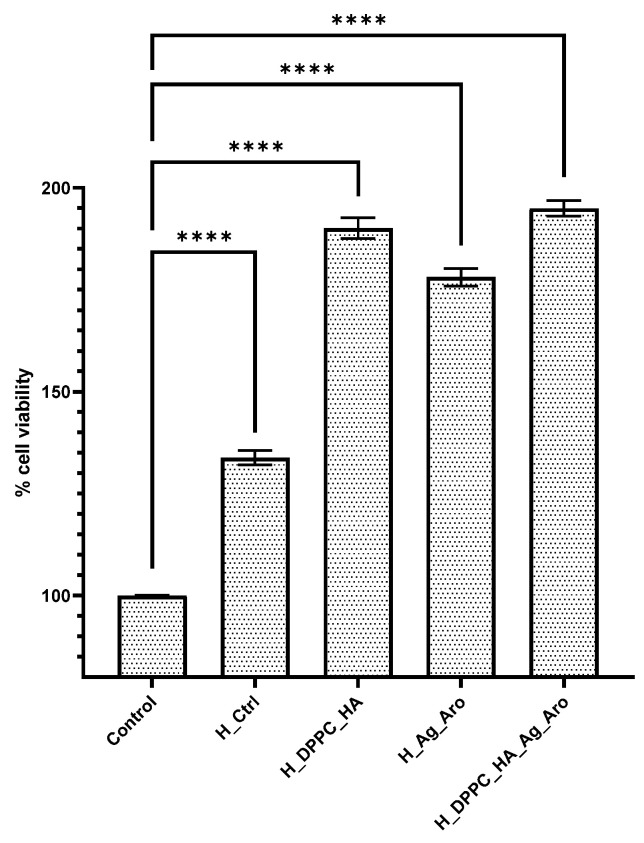
Graphical representation of human HaCaT keratinocytes viability after 24 h of contact with the tested samples (H_Ctrl, H_DPPC_HA, H_Ag_Aro, H_DPPC_HA_Ag_Aro). The represented data are the mean values of three independent experiments ± S.D. (**** *p* ≤ 0.0001 sample vs. control).

The results of the biological response evaluation after 24 h of incubation, expressed as cell viability in contact with the developed dressings, are shown in Figure 12. The use of polymers considered safe for skin contact was demonstrated by the cell viability value of approximately 130% recorded for the H_Ctrl formulation. Considering that the two polymeric matrices represent the main structural components of the dressings, this result is encouraging. The incorporation of active agents, such as DPPC_HA lipid vesicles, led to an increase in cell viability up to approximately 190%, which can be attributed to the proliferative properties of HA that promote cell growth and regeneration [23]. The H_Ag_Aro formulation showed a cell viability of around 180%, slightly lower than the H_DPPC_HA formulation. This reduction is most likely due to the presence of Ag_Aro nanoparticles, which can induce a more aggressive cellular response; however, cell viability and proliferation were still evident [74]. The formulation of particular interest, H_DPPC_HA_Ag_Aro, achieved cell viability values of up to 195%, confirming both its biocompatibility and its ability to support the development of new cells and, consequently, skin regeneration. The presence of DPPC_HA lipid vesicles in this formulation likely mitigates the cellular stress that may be induced by Ag_Aro nanoparticles [75]. Overall, all formulations maintained cell viability above 130% after 24 h of incubation, indicating promising biocompatibility and suitability for wound healing applications.

**Figure 13:**
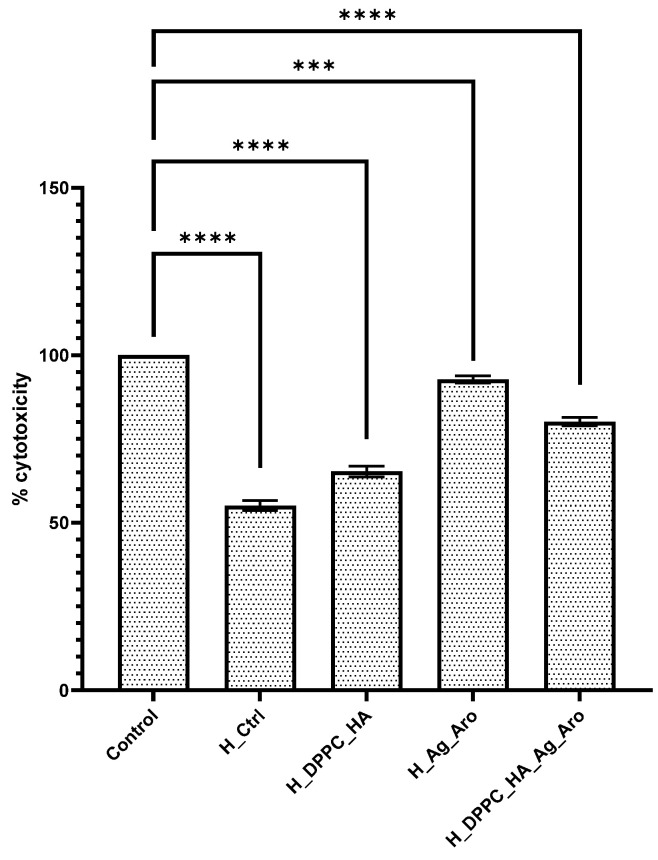
Graphical representation of the LDH leakage in the keratinocyte culture medium after 24 h of interaction with the tested samples as a measure of material cytotoxicity. The represented data are the mean values of three independent experiments ± S.D. (*** *p* ≤ 0.001, **** *p* ≤ 0.0001 sample vs. control).

The formulations were also evaluated in terms of their ability to preserve cell membrane integrity when in contact with the developed hydrogels, by measuring the LDH release after 24 h of incubation (Figure 13). Starting with the H_Ctrl sample, composed solely of the selected base polymers for the dressing matrices, a low level of cellular stress was observed, further confirming the biocompatibility of the ALG and PVA blend. The H_DPPC_HA formulation exhibited slightly higher values yet still maintained good biocompatibility. In contrast, the sample containing Ag_Aro nanoparticles (H_Ag_Aro) appeared to exert a more pronounced impact on the cells, leading to increased membrane disruption. This effect can be attributed to the intrinsic activity of the nanoparticles, which also display multiple morphologies. The dual active-agent formulation (H_DPPC_HA_Ag_Aro) confirmed, in this context as well, that the presence of DPPC_HA vesicles mitigates the cellular impact of Ag_Aro nanoparticles, resulting in lower cytotoxicity values compared to H_Ag_Aro alone. Overall, the 24-h results indicate cytotoxicity levels that support the potential use of these dressings within the range of biomaterials suitable for chronic wound healing applications.

## 3. Discussion

This study successfully developed a layered PVA–alginate hydrogel incorporating HA-loaded DPPC liposomes and AgNPs for regenerative wound therapy. The dual-layered design leveraged the structural robustness of PVA and the biocompatibility of alginate, while the integration of bioactive components provided synergistic benefits for wound healing.

Bilayer hydrogels are increasingly promising for wound care, as they mimic skin’s hierarchical structure by combining a protective outer barrier (preventing infection and dehydration) with a bioactive inner layer (promoting cell adhesion and tissue regeneration) [22]. Among these systems, ALG and PVA blends stand out for their synergistic advantages, such as enhanced mechanical strength, tunable swelling, and cytocompatibility, which can be further optimized through cross-linking (e.g., with Ca^2+^) to improve physicochemical and biological performance [76].

Several studies have explored the use of layered/hybrid hydrogel systems toward creating advanced wound healing options. For instance, Bahadoran et al. [77] have developed an ALG/PVA scaffold loaded with PCL-based microspheres, which enabled sustained, burst-free delivery of basic fibroblast growth factor (bFGF). The scaffold contributed to accelerated wound closure in vivo without compromising biocompatibility or hydrogel integrity. Alternatively, Estrada-Villegas and colleagues [62] reported the development of an ALG/PVGA (a PVA-derived polymer) hydrogel loaded with AgNPs using radiation-induced synthesis. This system demonstrated promising swelling behavior in various media, sustained AgNP stability over a one-month period, and progressive biodegradation in soil over 120 days. Cytotoxicity assays on NCTC 929 cells confirmed the material’s biocompatibility.

Other research groups have investigated the association of PVA with different natural polymers. Barba et al. [78] have fabricated gelatin–PVA bilayer hydrogels via radiation-induced cross-linking. This approach demonstrated functional layer-specific performance: gelatin promoted fibroblast proliferation and enzymatic degradation, whereas PVA offered anti-adhesion characteristics, structural stability, and non-cytotoxicity. Electrospun-hydrogel bilayers, as shown by Kamali and Shamloo [79], also revealed synergistic mechanical reinforcement and enhanced tissue integration compared to single-layer systems. Specifically, the authors have reported a scaffold made of an electrospun PCL and PVA sheet layer and a porous hydrogel layer comprising chitosan and gelatin, which maintains adequate cell proliferation and integration in vivo, highlighting the clinical relevance of such mechanically reinforced systems.

In this context, our bilayer design leverages polymeric material advantages while incorporating dual bioactive components (DPPC_HA-liposomes and Ag nanoparticles) to address both regeneration and infection—a gap in prior ALG/PVA systems. FTIR and SEM analyses confirmed the successful integration of bioactive components without compromising the hydrogel’s structural integrity. The hydrogel exhibited a well-defined porous structure, critical for exudate absorption and gas exchange. The incorporation of DPPC_HA liposomes and Ag_Aro nanoparticles influenced swelling and degradation rates, with the combined formulation (H_DPPC_HA_Ag_Aro) showing balanced swelling and controlled degradation—optimal for prolonged wound contact. Hydrogel degradation is strongly dependent on environmental pH. Under acidic conditions, protonation of alginate carboxylate groups enhances hydrolysis and matrix destabilization [80,81], whereas alkaline environments promote chain scission and weaken the PVA–alginate network [77,82]. Consequently, degradation rates measured at pH 7.4 in this study reflect behavior under near-physiological conditions but may differ in wounds characterized by acidic or alkaline microenvironments.

Previous studies have shown that the incorporation of AgNPs into alginate-based hydrogels can enhance swelling capacity, which has been attributed to nanoparticle-induced restructuring of the polymer network and increased porosity, thereby facilitating water diffusion into the matrix [71,72].

A green synthesis approach was employed to produce Ag nanoparticles using Aronia powder as a reducing agent. The phytochemicals present in Aronia are assumed to contribute both reducing and stabilizing functions. In the green synthesis of silver nanoparticles using Aronia powder, the compounds responsible for the reduction of Ag^+^ ions to metallic Ag^0^ are mainly polyphenols, including flavonoids and anthocyanins, as well as phenolic acids and vitamin C. These bioactive molecules act as natural reducing agents by donating electrons, while also stabilizing the formed nanoparticles by binding to their surface [37,39,41,60,83].

The minimal LDH release observed with the H_Ag_Aro formulation may stem from the antioxidant-rich polyphenols present in Aronia extracts, which serve as natural capping/stabilizing agents around Ag nanoparticles, improving their cytocompatibility and reducing membrane stress even in the presence of silver nanoparticles [84,85]. Several studies revealed that encapsulation of Ag nanoparticles within DPPC-based liposomes creates a lipidic microenvironment that significantly reduces ROS generation and caspase-3 activation in keratinocyte cultures, compared to free Ag nanoparticles, thereby mitigating membrane stress and supporting higher cell viability [75,86]. Even though we did not use liposomes for Ag nanoparticle encapsulation, this may represent a promising direction for future research.

In our study, DPPC-based liposomes were employed for HA encapsulation. DPPC_HA liposomes showed larger hydrodynamic diameters (~1608 nm) than empty liposomes, confirming HA loading, and indicating significant swelling, surface hydration, or even mild aggregation in aqueous solution. Moreover, the observed high cell viability (~190%) supported HA’s proliferative role. Previous cellular uptake studies have demonstrated that HA-functionalized liposomes undergo CD44-mediated endocytosis, leading to enhanced internalization in CD44-expressing cells, even when the particles are negatively charged (as is the case with the DPPC liposomes developed in our study) [50,87,88].

DPPC_HA systems demonstrated enhanced colloidal stability and potential for targeted delivery, supported by their negative zeta potential and uniform distribution within the PVA matrix. Moreover, all hydrogel formulations showed excellent biocompatibility with HaCaT keratinocytes, as evidenced by high cell viability (>130%) in MTT assays and low cytotoxicity in LDH tests. The H_DPPC_HA_Ag_Aro formulation outperformed others, suggesting that the combined action of hyaluronic acid (pro-regenerative) and silver nanoparticles (antimicrobial) creates a conducive microenvironment for wound healing.

Interesting possibilities for wound healing have also been reported by using DPPC-based liposomes for the delivery of other therapeutic agents, or by involving a different lipid composition for HA encapsulation. For instance, Ni et al. [87] have utilized undecylenoyl-phenylalanine (UP) liposomes loaded with HA for efficacious transdermal drug delivery. In particular, UP liposomes loaded with low molecular weight HA have demonstrated skin hydration activity, strong interaction with keratin and lipid, good skin penetration and retention, and CD44 receptor-mediated endocytosis. On a different note, Fang and colleagues [89] have fabricated liposomes by intercalating DPPC with lycopene, loaded them with tobramycin, and further formulated them with HAMA hydrogel. Their developed liposomal-hydrogel systems exhibited antioxidative and antibacterial activities, promoted cell migration and angiogenesis, improved re-epithelization and collagen deposition, subsequently enhancing wound healing.

All in all, the herein proposed bilayer design spatially separates functionalities: the PVA layer promotes tissue regeneration via hyaluronic acid, while the alginate layer provides antimicrobial protection via Ag_Aro. This multifunctionality addresses key challenges in chronic wound management, including infection control and tissue repair. Nonetheless, future in vivo studies are needed to validate the therapeutic efficacy of the hydrogel, including its antimicrobial activity, wound closure rates, and long-term safety. Moreover, further optimization of component ratios could refine performance for specific wound types.

In conclusion, this layered hydrogel represents a promising advanced wound dressing candidate, combining structural versatility, bioactive delivery, and biocompatibility to address the complexities of chronic wound healing.

## 4. Materials and Methods

### 4.1. Materials

For the preparation of DPPC-based liposomes, 1,2-dipalmitoyl-sn-glycero-3-phosphocholine (DPPC), chloroform, low molecular weight hyaluronic acid, and ultrapure water were used. DPPC was purchased from Avanti Polar Lipids (Sigma Aldrich/Merck, Burlington, MA, USA), while the other reagents were obtained from Sigma Aldrich/Merck (Burlington, MA, USA).

For silver nanoparticle green synthesis, silver nitrate (AgNO_3_), freeze-dried Aronia fruit powder, and ultrapure water were employed. AgNO_3_ was acquired from Sigma Aldrich/Merck (Burlington, MA, USA), while Aronia powder was procured from a local supplier in Romania.

For hydrogel fabrication, a combination of two polymers was selected: the synthetic polymer polyvinyl alcohol (PVA) and the natural polymer sodium alginate (ALG), both dissolved in ultrapure water. Both polymers were purchased from Sigma Aldrich/Merck (Burlington, MA, USA).

### 4.2. Synthesis of DPPC-Based Lipid Vesicles Loaded with Hyaluronic Acid

DPPC was dissolved in chloroform in a round-bottom flask, maintaining a 1:4 *w*:*v* ratio DMPC:CHCl_3_, similar to the method described in our previous study [27]. The dry lipid film was generated by subjecting the solution to rotary evaporation under the following operating parameters: water bath temperature at 40 °C, rotational speed at 80 RPM, and vacuum pressure at 500 mbar.

The lipid film was then hydrated with 20 mL of ultrapure water, and the flask was placed in an ultrasonic bath at 40 °C for 15 min to facilitate vesicle formation. This resulting dispersion constituted the DPPC stock solution, which was subsequently used to synthesize two types of liposomal formulations: DPPC_Ctrl (control liposomes encapsulating only water) and DPPC_HA (liposomes encapsulating hyaluronic acid). The DPPC_Ctrl sample was prepared by mixing ultrapure water and DPPC stock solution at a volume ratio of 2:1 (*v*:*v*). For the DPPC_HA sample, the same ratio was used, with the addition of hyaluronic acid dissolved in the ultrapure water before mixing, resulting in a final hyaluronic acid concentration of 5 mg/mL. This concentration was selected to provide adequate HA availability for CD44-mediated interactions while preserving vesicle formation and workable viscosity. Similar HA levels have been reported in HA/DPPC mixtures and HA-modified liposomal systems designed for dermal/ocular application, supporting both bioactivity and formulation stability [87,90].

Each solution was subjected to probe ultrasonication to ensure the formation of small-sized liposomes with the following settings: 20% amplitude, a total sonication time of 5 min, applied in 10-s on/5-s off cycles. After sonication, the dispersions were transferred into centrifuge tubes, subjected to physicochemical analysis, and stored at room temperature.

### 4.3. Green Synthesis of Silver Nanoparticles Using Aronia Powder

The synthesis began with the preparation of the Aronia extract. A 5-g amount of freeze-dried Aronia powder was dispersed in 200 mL of ultrapure water, heated to 60 °C, and stirred magnetically for 30 min. The mixture was then allowed to cool to room temperature and subsequently centrifuged to collect the supernatant, which served as the reducing agent for the green synthesis of silver nanoparticles.

In parallel, a silver nitrate precursor solution was prepared by dissolving AgNO_3_ in ultrapure water to a final concentration of 10 mM. A volume of 100 mL of Aronia extract was transferred into a Berzelius beaker and shielded from light using aluminum foil, as the reduction reaction requires dark conditions. The AgNO_3_ solution was added dropwise while stirring continuously. Once the addition was complete, stirring was stopped, and the beaker was fully covered with aluminum foil. It was then incubated in an oven at 60 °C for 12 h to allow the silver ion reduction to proceed.

After the reaction, the mixture was centrifuged to collect the silver nanoparticle pellet. The pellet was washed five times with ultrapure water until the supernatant became fully transparent, indicating the removal of residual Aronia extract. The purified pellet was then dried in an oven at 60 °C for 12 h, yielding a powder referred to as Ag_Aro, which was subsequently subjected to physicochemical characterization.

### 4.4. Fabrication and Layering of Hydrogel-Based Wound Dressings

Following the successful synthesis and characterization of DPPC-based liposomes and Ag_Aro nanoparticles, these nanostructures were incorporated into hydrogel matrices aimed at applications in skin regeneration, particularly in the treatment of chronic wounds.

The hydrogels were designed in a bilayer configuration using two polymer solutions prepared for hydrogel fabrication: 5% PVA solution and 5% ALG solution.

Four types of bilayer hydrogel dressings were fabricated using a layer-by-layer casting method, with each layer subjected to a freezing step immediately after casting. All formulations consist of a polyvinyl alcohol (PVA) layer and a sodium alginate (ALG) layer in a 1:1 volume ratio, with or without the incorporation of active agents. The control sample (H-Ctrl) contains only PVA and ALG, without any bioactive compounds. In the H-DPPC-HA formulation, DPPC-HA lipid vesicles were incorporated into the PVA layer at a final concentration of 20% (*v*/*v*), while the second layer consisted solely of ALG. The H-Ag-Aro sample featured a PVA layer without lipids and an ALG layer supplemented with Ag_Aro nanoparticles at a final concentration of 1 mg/mL. The H-DPPC-HA-Ag-Aro sample combined both active agents: the PVA layer contained 20% (*v*/*v*) DPPC-HA vesicles, and the ALG layer included 1 mg/mL of Ag_Aro nanoparticles.

**Scheme 1:**
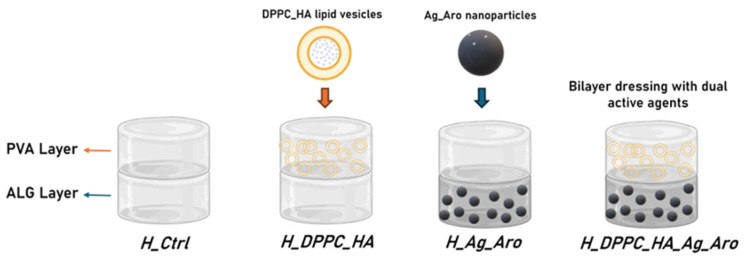
Schematic representation of the four developed bilayer hydrogel dressings: H_Ctrl—PVA first layer and ALG second layer without active agents; H_DPPC_HA—PVA layer containing DPPC_HA lipid vesicles and ALG layer without active agents; H_Ag_Aro—PVA layer without active agents and ALG layer containing Ag_Aro nanoparticles; H_DPPC_HA_Ag_Aro—PVA layer containing DPPC_HA lipid vesicles and ALG layer containing Ag_Aro nanoparticles.

Several formulations were developed to isolate and assess the individual contribution of each active component, as illustrated in Scheme 1.

### 4.5. Characterization Methods

#### 4.5.1. X-Ray Diffraction (XRD)

The crystallinity and crystal parameters of the silver nanoparticles were assessed through an X-ray diffraction technique, using a PANalytical Empyrean model diffractometer purchased from PANalytical (Almelo, The Netherlands), equipped with a hybrid monochromator (2×Ge 220) on the incident side and a parallel plate collimator mounted on a PIXcel 3D detector on the diffracted side. Grazing Incidence X-ray Diffraction (GIXRD) measurements were performed at room temperature, with an angle of incidence ω = 0.5° for Bragg angle values of 2θ between 10° and 80°, using Cu Kα radiation with λ = 1.5406 Å, a current of 40 mA, and a voltage of 45 kV.

#### 4.5.2. Scanning Electron Microscopy (SEM)

To investigate the morphological properties of silver nanoparticles and alginate-based hydrogels, SEM analysis was performed. The samples were fixed to a carbon-coated slide and placed in the analysis chamber of an Inspect F50 scanning electron microscope, purchased from Thermo Fisher—FEI (Eindhoven, The Netherlands). Images were obtained by recording the resulting secondary electron beam and electron beam scattering with an energy of 30 keV.

#### 4.5.3. Dynamic Light Scattering (DLS)

DLS analyses were performed on a DelsaMax Pro type device (Beckman Coulter, Brea, CA, USA) equipped with a 532 nm laser. The powder samples were dispersed in ultrapure water at ambient temperature by subjecting them to ultrasound treatment for 10 min, using an ultrasonic bath.

#### 4.5.4. Fourier Transform Infrared Spectroscopy (FTIR)

Fourier transform infrared spectroscopy was used to identify the functional groups present in the samples. For this purpose, a Thermo iN10-MX FTIR spectrometer, purchased from Thermo Fisher Scientific, Waltham, MA, USA, and equipped with a ZnSe crystal, was utilized to collect spectra between 4000 and 400 cm^−1^ wavenumbers.

#### 4.5.5. Swelling Rate

To evaluate their ability to absorb fluid, the freeze-dried hydrogel samples were sectioned into cylindrical shapes with a diameter of 5 mm. Each cylinder was placed in 1 mL of phosphate-buffered saline (PBS, pH 7.4) to replicate physiologically relevant ionic and pH conditions. The degree of swelling was quantified using Equation (1).

(1) $$\mathrm{Swelling ratio}=\frac{W_{t}-W_{i}}{W_{i}}\times 100\%$$

where W_i_ corresponds to the initial mass of the dry sample and W_t_ denotes the mass obtained after immersion in PBS at the specified time point.

#### 4.5.6. Degradation Rate

The rate of hydrogel degradation was determined by analyzing the samples after a 72-h incubation in PBS. The percentage of mass loss was calculated according to Equation (2).

(2) $$Degradation=\left(1-\frac{W_{0}-W_{t}}{W_{0}}\right)\times 100\%$$

where W_0_ corresponds to the initial mass of the dry sample and W_t_ corresponds to the remaining dry mass after 72 h of incubation in PBS.

#### 4.5.7. Biological Evaluation

The biocompatibility of synthesized dressings was evaluated using XTT reagent (2,3-Bis-(2-Methoxy-4-Nitro-5-Sulfophenyl)-2H-Tetrazolium-5-Carboxanilide) according to the manufacturer’s protocol (CyQUANT™ XTT Cell Viability Assay Kit, Thermo Fischer Scientific, Waltham, MA, USA). The assay kit includes the XTT reagent and an Electron Coupling Reagent. The XTT reagent is a tetrazolium-based compound sensitive to cellular redox potential. Actively viable cells convert the water-soluble XTT compound to an orange-colored formazan product. The sensitivity and consistency of the assay are significantly increased when used with the Electron Coupling Reagent. The human keratinocyte HaCaT was grown in DMEM medium (Sigma-Aldrich, Saint Luis, MO, USA) supplemented with 10% fetal bovine serum, 1% antibiotics (penicillin and streptomycin) (Sigma-Aldrich, Saint Luis, MO, USA), and changed twice a week. The cells were placed in 96-well plates at a density of 3000 cells/well in the presence of double-layered dressings for 24 h and 48 h. The control samples were represented only by cells cultivated in the same conditions but without the dressings. Subsequently, 70 µL of XTT solution was added to the cells, followed by incubation at 37 °C for 4 h. After vigorous homogenization of formazan crystals, the absorbance was read at 450 nm using a spectrophotometer.

The cytotoxicity was evaluated using the LDH Cytotoxicity Assay Kit (Invitrogen™ CyQUANT™ Thermo Fischer Scientific, Waltham, MA, USA). Quantification of LDH concentration in media is an indicator of cellular cytotoxicity, so the assay can be used to monitor cytotoxicity from the same sample over time. Lactate dehydrogenase (LDH) is a cytosolic enzyme present in many different cell types, and the damage to the plasma membrane releases LDH into the surrounding cell culture media. The extracellular LDH in the media can be quantified by a coupled enzymatic reaction in which LDH catalyzes the conversion of lactate to pyruvate via NAD+ reduction to NADH. Oxidation of NADH by diaphorase leads to the reduction of a tetrazolium salt (INT) to a red formazan product that can be measured spectrophotometrically. The level of formazan formation is directly proportional to the amount of LDH released into the medium, which is indicative of cytotoxicity. To perform the LDH cytotoxicity assay, an aliquot of 50 μL cell culture media is transferred to a new plate, and 50 μL of LDH reaction mixture is added. After a 30-minute incubation at room temperature, the assays are stopped by adding 50 μL of Stop Solution. Then fluorescence is measured using a microplate reader at 560 nm excitation and emission of 590nm. Statistical analyses were performed using GraphPad Prism version 9 (GraphPad Software, San Diego, CA, USA). Data are presented as mean ± standard deviation (S.D.) from three independent experiments. One-way analysis of variance (one-way ANOVA) was applied, followed by Tukey’s multiple comparisons post hoc test. Differences were considered statistically significant at *p* < 0.05. Significance levels were indicated as follows: *p* < 0.001 (***), and *p* < 0.0001 (****).

## 5. Conclusions

This study presents the design and characterization of a novel bilayer hydrogel system comprising PVA and ALG layers embedded with hyaluronic acid-loaded liposomes (DPPC_HA) and green-synthesized silver nanoparticles (Ag_Aro) for potential application in regenerative wound therapy. By combining the structural and mechanical advantages of synthetic PVA with the biocompatibility and gel-forming capacity of alginate, the resulting hydrogel offers an optimized environment for tissue repair. The layered configuration enabled the spatial separation of regenerative and antimicrobial functionalities, addressing the dual clinical needs for infection control and tissue repair in chronic wounds. The incorporation of DPPC_HA liposomes enhanced the regenerative potential of the PVA layer, while the addition of Ag_Aro nanoparticles enables antimicrobial protection, both critical features for the management of chronic wounds.

The hydrogel formulations exhibited favorable swelling profiles, structural stability, and in vitro cytocompatibility. Notably, the swelling behavior was modulated by the inclusion of nanostructured agents, reflecting their influence on network porosity and hydration dynamics.

Overall, the formulated hydrogel represents a promising multifunctional platform for advanced wound care, combining structural support, antimicrobial activity, and regenerative potential. While this work lays a foundation for future innovations in multifunctional wound dressings, further biological validation will be essential to confirm its clinical applicability and therapeutic effectiveness.

## Data Availability

Available from the authors by request. The cell line was obtained from American Type Culture Collection company (ATCC, Manassas, VA, USA).
